# Kinetin modulates physio-hormonal attributes and isoflavone contents of Soybean grown under salinity stress

**DOI:** 10.3389/fpls.2015.00377

**Published:** 2015-06-01

**Authors:** Muhammad Hamayun, Anwar Hussain, Sumera Afzal Khan, Muhammad Irshad, Abdul Latif Khan, Muhammad Waqas, Raheem Shahzad, Amjad Iqbal, Nazif Ullah, Gauhar Rehman, Ho-Youn Kim, In-Jung Lee

**Affiliations:** ^1^Department of Botany, Abdul Wali Khan University MardanMardan, Pakistan; ^2^Center of Biotechnology and Microbiology, University of PeshawarPeshawar, Pakistan; ^3^UoN Chair of Medicinal Plants and Marine Natural Products, University of NizwaNizwa, Oman; ^4^School of Applied Biosciences, College of Agriculture and Life Science, Kyungpook National UniversityDaegu, South Korea; ^5^Department of Agriculture, Abdul Wali Khan University MardanMardan, Pakistan; ^6^Department of Biotechnology, Abdul Wali Khan University MardanMardan, Pakistan; ^7^Department of Zoology, Abdul Wali Khan University MardanMardan, Pakistan; ^8^Department of Plant Sciences, University of California DavisDavis, CA, USA

**Keywords:** kinetin, soybean, salt stress, gibberellins, abscisic acid, jasmonic acid, salicylic acid

## Abstract

Crop productivity continues to decline due to a wide array of biotic and abiotic stresses. Salinity is one of the worst abiotic stresses, as it causes huge losses to crop yield each year. Kinetin (Kn) has been reported as plant growth regulator since long, but its role in improving plant growth and food quality under saline conditions through mediating phytohormonal cross-talk is poorly studied. Current study was designed to evaluate the impact of exogenously applied Kn on growth, isoflovones and endogenous phytohormones of soybean grown under NaCl induced salt stress. Soybean plants were grown in perlite (semi hydroponic), and under controlled green-house conditions. Elevated levels of exogenous Kn significantly mitigated the adverse effect of NaCl and rescued plant growth attributes, i.e., plant height, fresh and dry biomass of soybean plants grown in all treatments. Higher diadzen, glycitin, and genistin contents were observed in plants treated with elevated Kn in the presence or absence of NaCl induce salt stress. The gibberellins (GAs) biosynthesis pathway was up-regulated by Kn as the bioactive GA_1_ and GA_4_ contents were significantly higher in Kn treated plants, as compared to control, while GAs level reduced in NaCl treated plants. Contrary to GAs, the abscisic acid contents declined with Kn but promoted in NaCl stressed soybean plants. The endogenous jasmonic acid and salicylic acid contents of soybean enhanced with elevated Kn application, but they showed an antagonistic response under salt stress. Current study supports the active role of Kn to ameliorate the adverse effects of salt stress on the growth and food quality of soybean. The favorable role of Kn toward soybean growth under salt stress may be attributed to its potential to modulate cross-talk between the various phytohormones involved in soybean growth and its resistance to salinity stress.

## Introduction

The arable land continues to decline globally, as it is rendered unfavorable for cultivation due to a wide array of environmental constraints. Salinity is a major agricultural problem that is responsible for low yield on approximately 33% (40 million hectares) the irrigated land on earth (Norlyn and Epstein, 1984). If lifted untreated, the problem will become more severe by affecting approximately 50% of the arable land by 2050 (Hussain et al., 2008). Saline soils are characterized by high levels of sodium (Na^+^) and chloride (Cl^-^) ions that interfere with the normal growth and yield of the plants cultivated in such soils. Induced ionic and osmotic imbalance is the common mechanism through which salt stress affects plant metabolism ultimately resulting abnormal growth. Under saline conditions, plant cell accumulate compatible osmolytes and consequently uptake additional water from surrounding, thereby buffering the immediate effect of salts (Bartels and Sunkar, 2005; Misra and Gupta, 2005; Verbruggen and Hermans, 2008). However, growth of leguminous plants particularly soybean is severely affected by salts (Pathan et al., 2007), where slow growth is associated with restricted root water uptake and abnormal cell physiology (Apse et al., 1999; Zhu, 2002).

Soybean is a vital source of human nutrition across the world. Soybean is mostly grown for oil production, while a smaller portion is directly used by humans. Soybean and its products (chungkookjang, doenjang, douchi, ganjang, miso, natto, and tofu) constitute an important part of daily diet in countries like China, Korea, and Japan. There is an increase in soybean consumption worldwide, due to nutritional value attributed to the presence of healthy isoflavone. Isoflavone is important in human health-care, such as cardiovascular diseases, menopausal symptoms, bone resorption, and breast, prostate, and colon cancers (Messina, 2000; Allred et al., 2004). Daidzein and genistein are two most important isoflavone isomers, and are regarded as phytoestrogens due to their favorable role in human health-care (Omoni and Aluko, 2005). The isoflavones are chemically categorized in to four subgroups, i.e., aglycones, glycosides, malonyl glycoside, and acetyl glycosides (Ososki and Kennelly, 2003).

Plants integrate environmental signals (such as salinity) and phytohormonal signals [such as abscisic acid (ABA), gibberellins (GAs), jasmonic acid (JA), and salicylic acid (SA)] during growth regulation. The plant hormones are present in very minute quantities, yet they are crucial for plant growth and development. For instance, GAs are vital for seed germination, stem elongation, leaf expansion, and flowering (Magome et al., 2004), while JA influences seed germination, root growth, senescence, fertility, and fruit ripening (Creelman and Mulpuri, 2002; Wasternack and Hause, 2002). Additionally, JA is an essential component of plant immunity in insect-driven wounding, pathogens, and abiotic stresses such as low temperature, salinity, and drought (Wasternack and Parthier, 1997). Another phytohormone, SA mediates plant responses to a wide range of stresses, enabling plants to survive in conditions of biotic and abiotic stresses including salt stress (Keskin et al., 2010). Additionally, ABA regulates several physiological processes in plants including photosynthesis, growth, nitrate metabolism, heat production, ethylene production and flowering (Hayat et al., 2010).

Different strategies have been working to promote plant growth and productivity under saline conditions. Improving plant tolerance to salinity through conventional plant breeding techniques are laborious, time consuming, and depend upon the existing genetic variability. In addition, many attempts have been made to overcome salinity stress, including proper management and exogenous application of plant growth regulators (Javid et al., 2011). Kinetin (Kn), a synthetic cytokinin (CK) has been previously reported for reprogramming higher plant performance under normal and saline conditions. Beside a long history of Kn applications for the alleviation of salt stress in plants, little has been known about its role in the modulation of physio-hormonal attributes and enhancement of nutritional quality of plants under salinity. It has been reported that application of Kn modulates phenolic metabolism of *Vigna sinensis* and *Zea mays* grown under salt stress (Nemat Alla et al., 2002). Salt stressed phenotype of salt sensitive plants is normally characterized by low level of endogenous CKs which indicates that CKs levels could be a limiting factor under stress conditions. This may be a possible explanation of the growth improvement in salt stressed chickpea by the exogenous application of Kn. However, exposure of facultative halophyte *Mesembryanthemum crystallinum* to salt stress caused no change in the endogenous levels of zeatin-type CKs (Thomas and Bohnert, 1993). It has been suggested that Kn scavenges free radicals or it may be involved in antioxidative mechanism related to the protection of purine breakdown (Chakrabarti and Mukherji, 2003). Using mutant *Arabidopsis*, it has been demonstrated that CKs receptors act as negative regulators in ABA signaling as well as in osmotic stress responses (Tran et al., 2007). However, very little is known on interaction of kinetine with phytohormones other than ABA under saline conditions. In current study, we investigate the effect of Kn and NaCl on the growth attributes and food quality of hydroponically grown soybean cv. Daewonkong. Furthermore, a hormonal cross-talk resulting from Kn and NaCl application is also studied.

## Materials and Methods

### Plant Material and Growth Conditions

Surface sterilized seeds of soybean cultivar Daewonkong were sown in perlite containing pots (15 L) and kept under greenhouse conditions. Sterilization was done with 5% solution of sodium hypochlorite (NaOCl) for 15 min followed by thorough rinsing with distilled H_2_O. Modified hoagland solution containing 5 mM KNO_3_, 1 mM NH_4_NO_3_, 0.5 mM KH_2_PO_4_, 5 mM Ca(NO_3_)_2_4H_2_O, 1.5 mM Fe-EDTA, 2 mM MgSO_4_.7H_2_O, and other micro-nutrient in their original concentrations were used as fertilizer (Yoon et al., 2009). Kn was applied in the elevated conc. of 0.5, 1, and 5 μM, while a single conc. of NaCl (100 mM) was applied to soybean after 17 days of sowing (17 DAS). Each pot contained three plants, and received a single dose of Kn (50 ml) and NaCl (400 ml) at 17 DAS. Soybean plants were harvested after 2 weeks of Kn and NaCl application. However, for phytohormonal analysis, one-third of plants per treatment were harvested after 24 h of Kn and NaCl treatment, immediately frozen in liquid nitrogen, and later stored in refrigerator (Sanyo-ultra low, Japan) at −80°C. The experiment was designed as randomized complete block design (RCBD), consisting of eight treatments and 15 replicates per treatment.

### Growth Attributes Analysis

Soybean growth attributes, viz., plant length, shoot and root fresh and dry weights were measured at the harvest time, while chlorophyll content of fully expanded leaves was analyzed with the help of chlorophyll meter (Minolta Co., Ltd, Japan) just before the harvest (Hamayun et al., 2010). For soybean growth analysis, 20 plants per treatment were randomly selected. The dry biomass was measured after drying the plants at 70°C for 48 h in an oven (Bohm, 1979).

### Isoflavones Analysis

Isoflavones content of soybean leaves were measured by following the already established standard protocols (Rostagno et al., 2003; Phommalth et al., 2008). Soybean leaves were crushed to powder form, and 0.2 g of this powder was added to 10 ml of 80% EtOH and kept for 1 h in Ultrasonic bath (Kodo Co., Korea) set at 50°C. The samples were then kept in a shaking incubator (150 rpm) at 50°C for 15 h and then filtered through 0.45 μm syringe filter. The filtered samples (10 μl) were injected using gradient solutions, viz., acetonitrile and 0.1% of acetic acid in water by using TOTALCHROM V6.2.0.0.1 system with LC instrument control (PerkinElmer Series 200, USA), and a COL-CHOICE C_18_ column 4.6 mm × 150 mm (5 μm) packed. Sample elution was done at a flow rate of 1.0 ml/min and flavones in the sample were determined by UV-absorption (Series 200 UV/Vis Detector) at 260 nm. Identification of the isoflavones were based on comparisons with retention times of internal standards, including daidzein, genistein, and genistin (Sigma Chemical Co, USA), as well as glycitin, daidzin, 6′-0-actlygnistin, 6′-0-malonygenistin, and 6′-0-actyldaidzin (LC Laboratory, USA).

### Analysis of Endogenous Phytohormones

#### Gibberellins Analysis

Soybean shoot samples were lyophilized and then crushed to powder form. The powdered sample (0.5 g) was used for extraction and quantification of bioactive GA_1_ and GA_4_, following an established protocol (Lee et al., 1998). The GC (Hewlett-Packard 6890, 5973N mass selective detector) was equipped with HA-1 capillary column (30 m × 0.25 mm i.d. 0.25 μm film thickness), and oven temperature was programmed at 60°C for 1 min, then a rise of 15°C min^-1^ to 200°C, followed by rise of 5°C min^-1^ to 285°C. Helium carrier gas was kept at a head pressure of 30 kPa. The GC was directly interfaced to a mass selective detector with an interface and source temperature of 280°C, an ionizing voltage of 70 eV, and a dwell time of 100 ms. Full scan mode (the first trial) and three major ions of the supplemented (^2^H_2_) GAs internal standards (the second trial), and the endogenous GAs were monitored simultaneously (standard GAs were purchased from Prof. Lewis N. Mander, Australian National University, Canberra, ACT, Australia). The endogenous GA_1_ and GA_4_ contents were calculated from the peak area ratios of 506/508 and 284/286, respectively. The data was calculated in nano-grams per gram dry weight and the analysis was repeated three times.

#### Salicylic Acid (SA) Analysis

The endogenous free SA contents of soybean shoots were extracted and quantified as described (Enyedi et al., 1992; Seskar et al., 1998). Freeze-dried leaves (0.1 g) were ground to fine powder under liquid nitrogen and subjected to extraction sequentially done with 90 and 100% methanol (MeOH) by centrifuging at 10,000 × *g*. The pooled MeOH fractions dried under vacuum and the pellet was resuspended in 2.5 ml of 5% trichloroacetic acid. For further fractionation, ethyl acetate (EtOAc)/cyclopentane/isopropanol (49.5:49.5:1, v/v) was added and the free SA containing top organic layer was dried under nitrogen gas in a 4 ml vial. It was re-suspended in 1 mL of 70% MeOH. Determination of SA was performed on HPLC (Shimadzu, Japan) equipped with fluorescence detector (Shimadzu RF-10AXL, excitation and emission detected at 305 and 365 nm, respectively) and C18 reverse-phase column (HP hypersil ODS; particle size, 5 μm; pore size, 120-Å water). Sample was eluted at a flow rate of 1.0 ml/min.

#### Abscisic Acid (ABA) Analysis

Leave were ground to fine powder in liquid nitrogen for extraction of ABA as described earlier (Yoon et al., 2009). To the powder, 30 ml of extraction solution containing 95% isopropanol, 5% glacial acetic acid, and 20 ng of [(±)–3,5,5,7,7,7–d6]–ABA were added. After proper mixing and filtration the filtrate was dried by a rotary evaporator. The residue was taken in 4 ml of 1 N sodium hydroxide solution and washed three times with 3 ml of methylene chloride for the removal of lipophilic materials. After setting pH of the aqueous phase to 3.5 with 6 N hydrochloric acid, it was partitioned with EtOAc three times. Pooled EtOAc extracts were then evaporated and the dried residue was dissolved in phosphate buffer (pH 8.0). The sample was then passed through a polyvinylpolypyrrolidone (PVPP) column. Again, pH of the phosphate was adjusted to 3.5 with 6 N HCl and partitioned three times into EtOAc. After evaporating the pooled fractions to dryness, the residue was dissolved in dichloromethane (CH_2_Cl_2_) and passed through a silica cartridge (Sep-Pak; Water Associates, Milford, MA, USA) pre-washed with 10 ml of diethyl ether: MeOH (3:2, v/v) and 10 ml of dichloromethane. ABA was eluted from the cartridge with 10 ml of diethyl ether (CH_3_–CH_2_)_2_O: MeOH (3:2, v/v). The extracts were dried and methylated by adding diazomethane for GC/MS-SIM (6890 N network GC system, and the 5973 network mass-selective detector; Agilent Technologies, Palo Alto, CA, USA) analysis. For quantification, the Lab-Base (ThermoQuset, Manchester, UK) data system software was used to monitor responses to ions with an m/e of 162 and 190 for Me-ABA and 166 and 194 for Me-[^2^H6]-ABA.

#### Jasmonic Acid (JA) Analysis

The endogenous JA contents of soybean shoots were determined by following a modified version of the protocol of McCloud and Baldwin (1997). Fine powder made by grinding the lyophilized samples (0.1 gm) was suspended in a solution of acetone and 50 mM citric acid (70:30, v/v), containing [9,10-^2^H_2_]-9,10-dihydro-JA (20 ng) as an internal standard. The suspension was kept overnight under room temperature for slow evaporation necessary for avoiding the loss of fatty acids of volatile nature. On the next day, the resultant aqueous solutions were filtered and the filtrate was extracted three times with 10 ml of diethyl ether. Solid phase extraction cartridge (500 mg of sorbent, aminopropyl) was loaded with the pooled extracts and washed with 7 ml of trichloromethane and 2-propanol (2:1, v/v). Elution of the bound JA and the relevant standard was carried out with a mixture of 10 ml of diethyl ether and acetic acid (98:2, v/v). The residue obtained after evaporation of solvents was esterified with excess diazomethane and its volume was brought to 50 μl with dichloromethane. Quantification of JA was done by GC/MS (6890N network GC system, and 5973 network mass selective detector; Agilent Technologies, Palo Alto, CA, USA). To enhance the sensitivity of the method, spectra were recorded in the selected ion mode, i.e., in case of JA determination, monitored the fragment ion at *m/z* = 83 amu corresponding to the base peaks of JA and [9, 10-^2^H_2_]-9, 10-dihydro-JA (Koch et al., 1999). Amount of JA was calculated and represented as nano-grams per gram dry weight. Experiment was repeated three times.

#### Statistical Analysis

Sigma Plot software (2004) was used to calculate SD and SE using of the data. The mean values were compared using Duncan’s multiple range tests at *P* < 0.05 (ANOVA SAS release 9.1; SAS, Cary, NC, USA).

## Results

### Kinetin Promotes Plant Growth and Mitigates Salinity Stress

**FIGURE 1:**
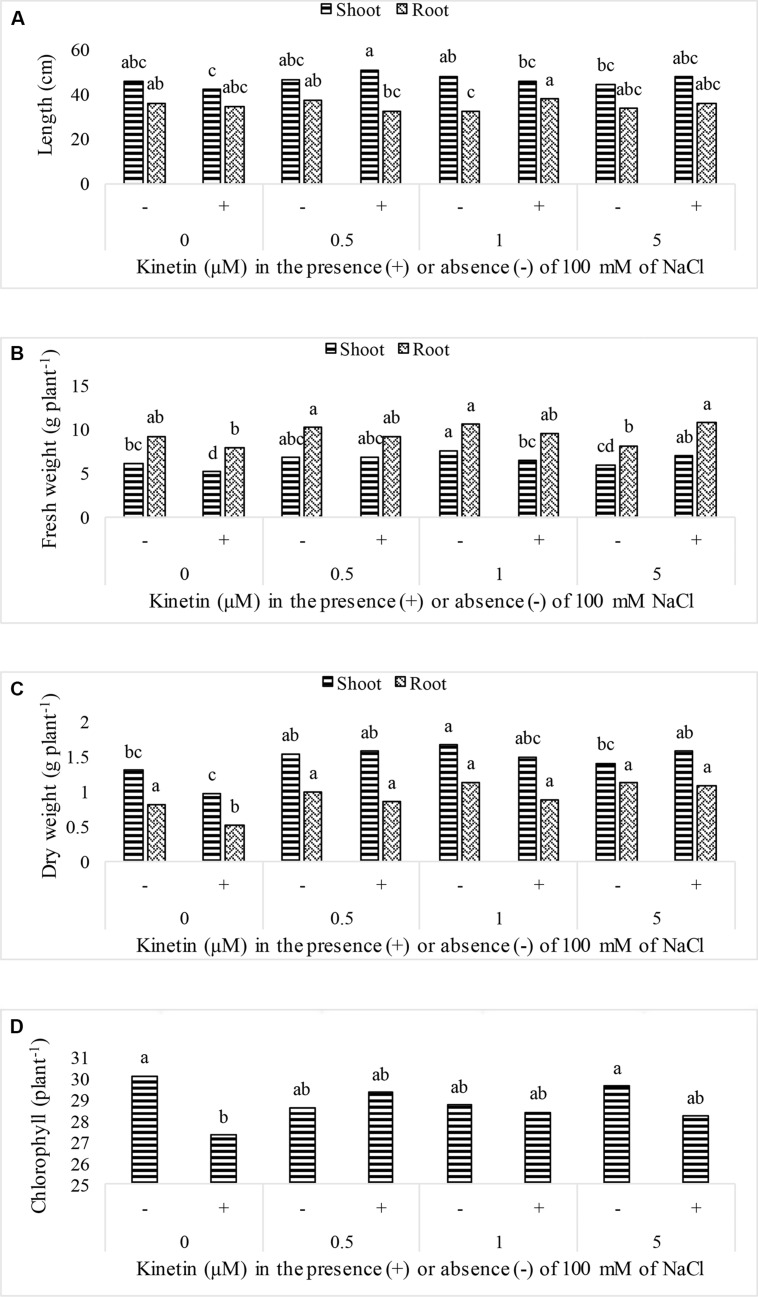
**Effect of different doses of Kn in the presence of absence of 100 mM NaCl on; (A) plant length, **(B)** fresh weight, **(C)** dry weight, and **(D)** chlorophyll contents of soybean cv. Daewonkong.** Bars labeled with common letter(s) are not significantly different at the 5% level by DMRT.

Soybean growth was significantly promoted by elevated Kn levels and rescued plant growth under NaCl salt stress. Maximum shoot length (90.92 cm) was recorded for plants that received combined dose of Kn (0.5 μM) and NaCl, while highest shoot fresh biomass (7.57 gm), shoot dry biomass (1.66 gm), root fresh biomass (10.53 gm), and root dry biomass (1.13 gm) were observed in Kn (1 μM) treated plants (**Figure 1**). The chlorophyll contents were not much affected by Kn and NaCl application. Sole application of NaCl greatly obstructed growth attributes of soybean. The chlorophyll contents of soybean leaves significantly declined under stress conditions as compared to control. Current findings suggest that Kn application enhanced soybean growth by alleviating the negative effect of NaCl (**Figure 1**).

### Kinetin Improved Soybean Isoflavone Contents

**FIGURE 2:**
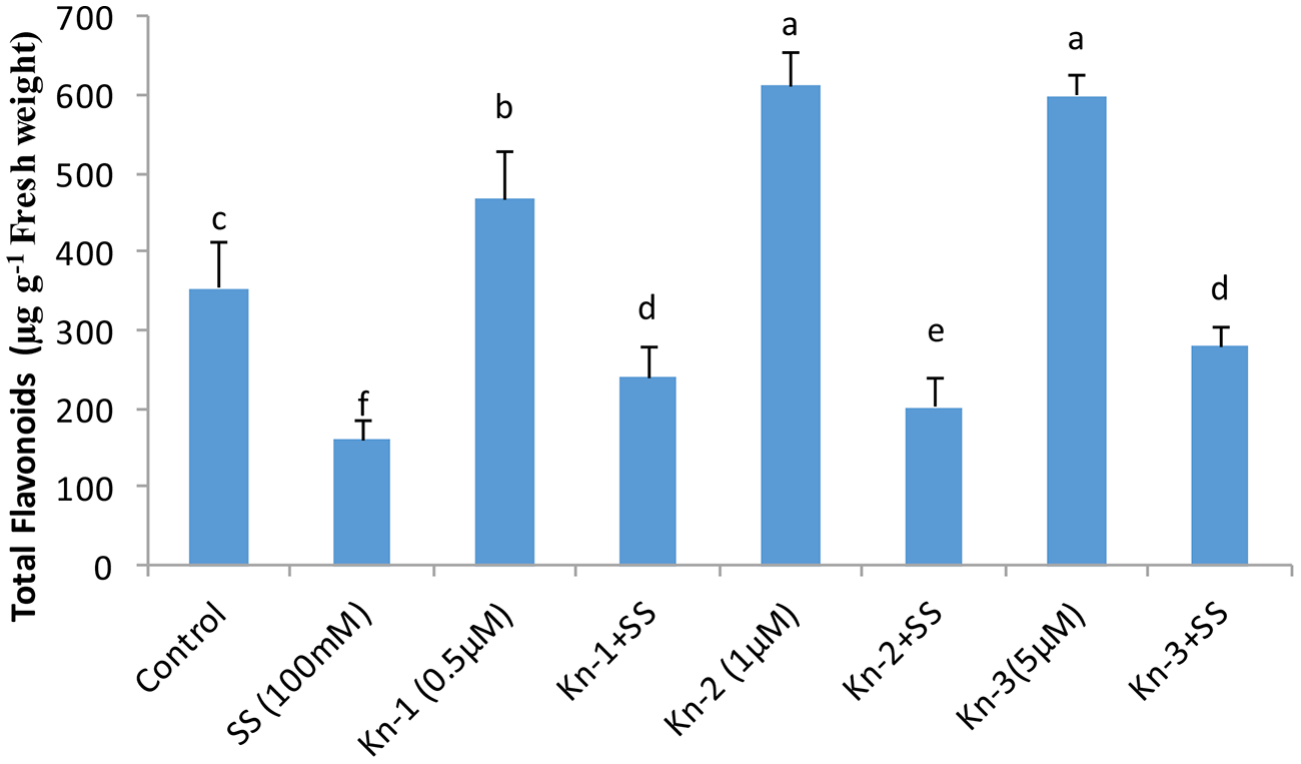
**Effect of Kn and salt stress on the concentrations of total flavonoids in soybean cv. Daewonkong**. Bars in the graph represent means of three replicated with ±SEM. Bars labeled with same letter are not significantly different at *P* > 0.05 by Duncan Multiple Range Test (DMRT).

**FIGURE 3:**
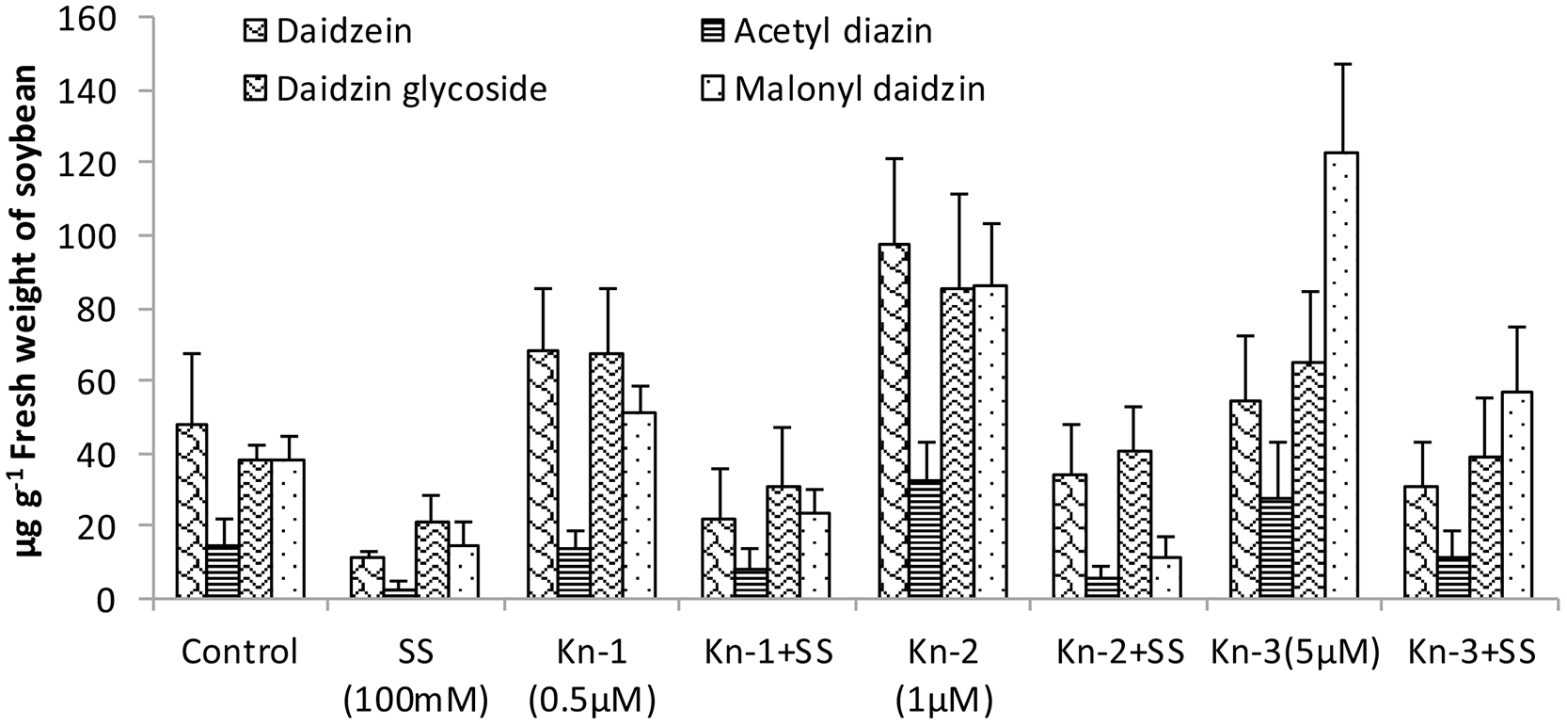
**Effect of Kn and salt stress on the concentrations of aglycones, acetyl glycosides, glycosides and malonyl glycosides of daizein in soybean cv. Daewonkong**. Bars in the graph represent means of three replicated with ±SEM.

**FIGURE 4:**
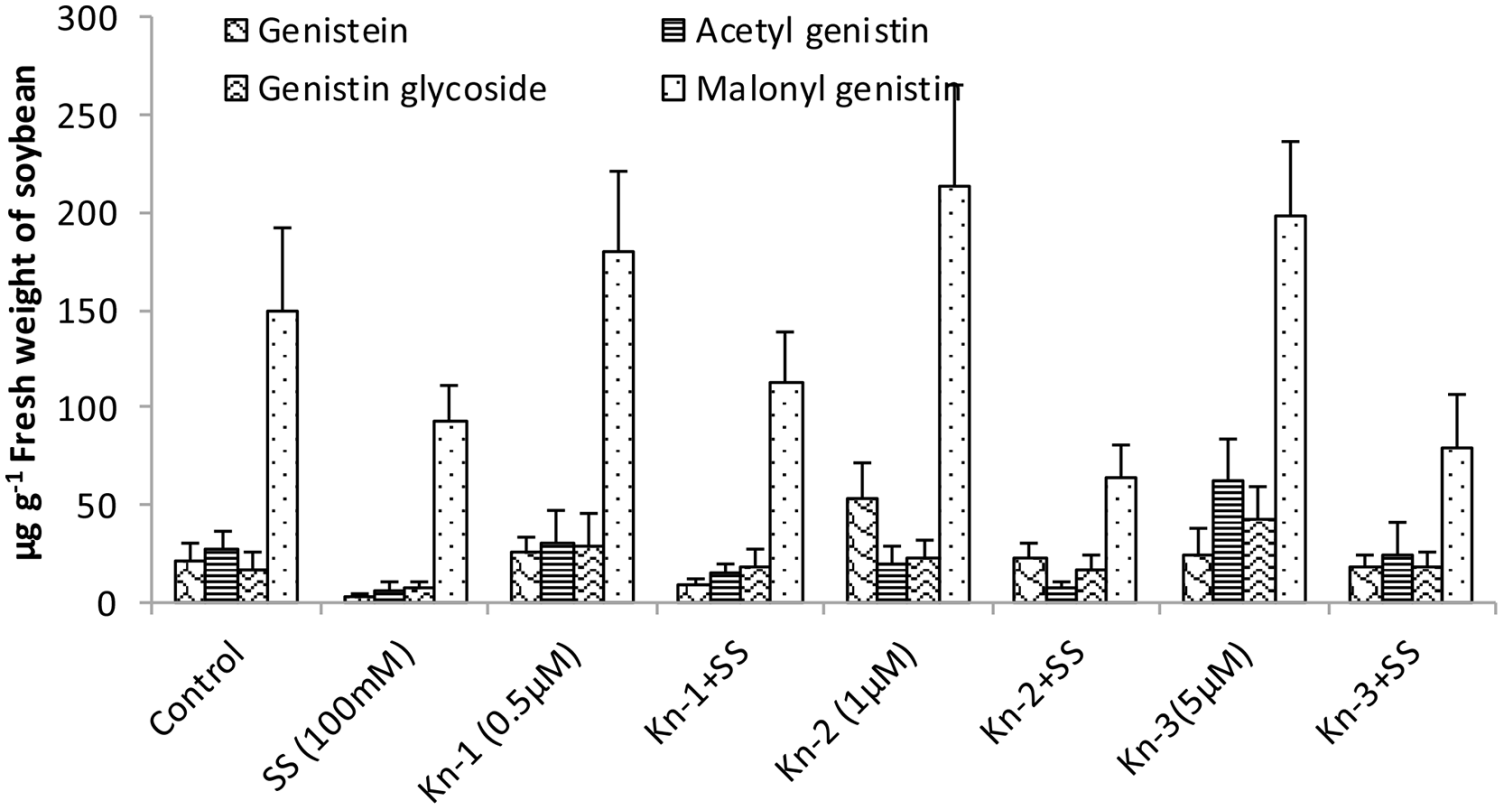
**Effect of Kn and salt stress on the concentrations of aglycones, acetyl glycosides, glycosides and malonyl glycosides of genistin in soybean cv. Daewonkong**. Bars in the graph represent means of three replicated with ±SEM.

Isoflavones analysis showed that both Kn and salt stress significantly affected their levels in soybean leaves (**Figure 2**). Salt stress significantly decreased the total isoflavones, while Kn effectively improved the quantities of isoflavones, and mitigated the negative impact of NaCl induced salt stress on isoflavone biosynthesis. Maximum isoflavone content (610.9 μg/g) was found in Kn (1 μM) treated plant, while least (159.8 μg/g) in NaCl treated plants, as compared to 354.3 μg/g in control (**Figure 2**). Similarly, maximum quantities of the highly valued phytoestrogens (daidzein and genistein) were observed in Kn (1 μM) treated plants (**Figures 3** and **4**). Current study reports the favorable role of Kn on the isoflavones content of soybean, and suggest that Kn counteracted the negative effect of the NaCl induced salt stress on isoflavones biosynthesis. It was also observed that daidzein was more abundant than genistein in the soybean (**Figures 3** and **4**).

### Kinetin Stimulates Endogenous Gibberellins Biosynthesis in Soybean

**FIGURE 5:**
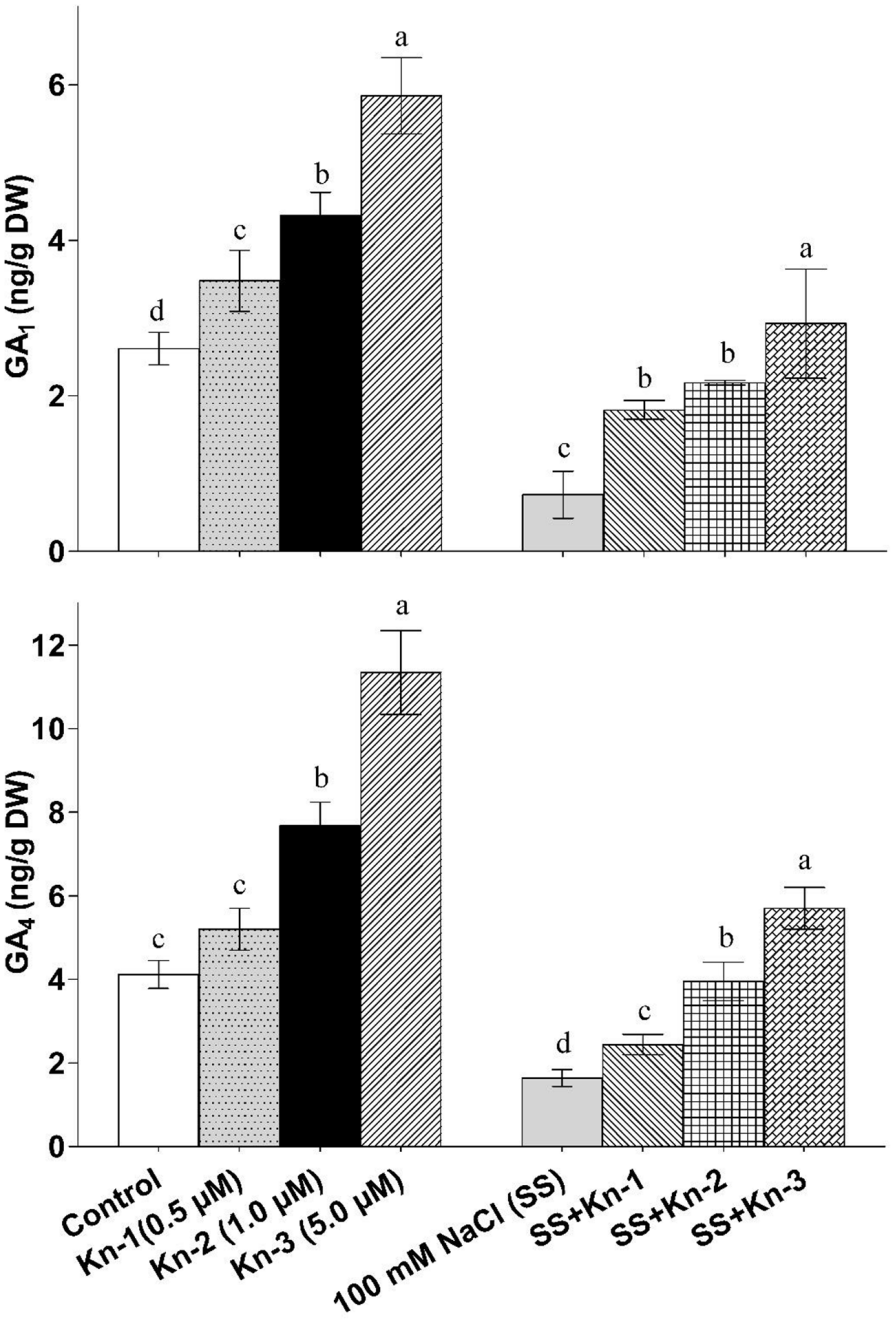
**Effect of exogenous application of Kinetin (Kn) and NaCl on the endogenous bioactive Gibberellins (GAs) including GA_1_ and GA_4_ contents of soybean shoots**. Seedlings of soybean were exposed to different concentrations of Kn and NaCl after 17 days of sowing (17 DAS) and harvested after 24 h. Graph represents data from nine replicates in two independent experiments (data are mean ± SD; *n* = 18). Bars labeled with different letters shows significant difference (*p* < 0.05; Duncan test).

Kinetin application significantly enhanced bioactive GA_1_ and GA_4_ contents of soybean, while salt stress markedly reduced biosynthesis of bioactive GAs. The bioactive GAs (GA_1_ and GA_4_) levels increased with elevated Kn application as maximum GA_1_ (5.86 ng/g) and GA_4_ (11.34 ng/g) contents were found in plants that received 5 μM Kn (see **Figure 5**). On the other-hand NaCl treated plants contained significantly reduced GA_1_ (0.73 ng/g) and GA_4_ (1.64 ng/g) contents, while plants treated with both Kn and NaCl showed considerable recovery as compared to sole NaCl treatments (**Figure 5**). Current finding clearly suggests that Kn stimulates GA biosynthesis in soybean under saline conditions.

### Kinetin Stimulates Free Salicylic Acid Biosynthesis in Soybean

**FIGURE 6:**
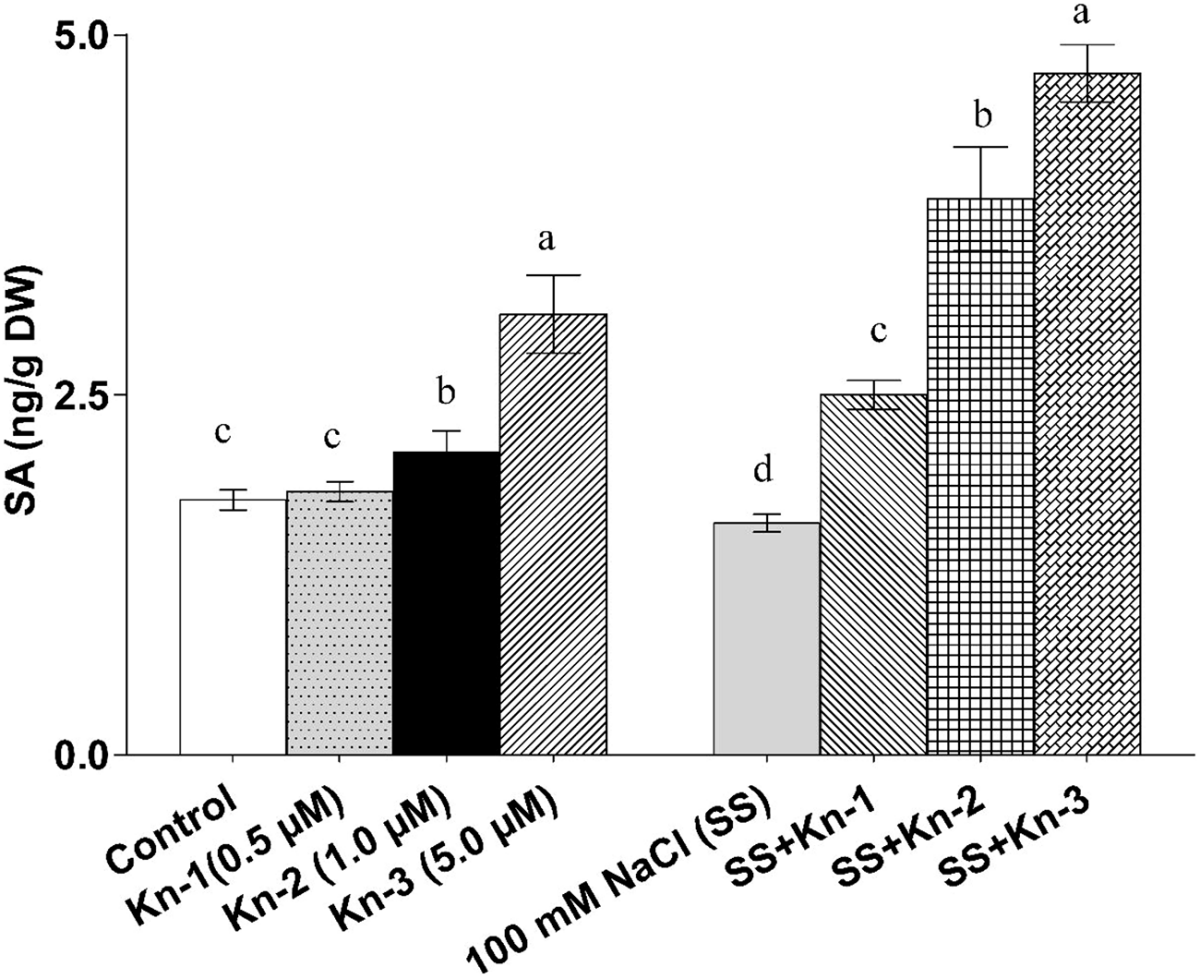
**Effect of exogenous application of Kn and NaCl on the endogenous level of free [Salicylic acid (salicylic acid)] SA of soybean shoots**. Seedlings of soybean were exposed to different concentrations of Kn and NaCl after 17 days of sowing (17 DAS) and harvested after 24 h. Graph represents data from nine replicates in two independent experiments (data are mean ± SD; *n* = 18). Bars labeled with different letters shows significant difference (*p* < 0.05; Duncan test).

The endogenous free SA contents of soybean significantly increased with the addition of elevated Kn but markedly decreased in sole NaCl treated plants. However, an addition of Kn to NaCl treatments showed a significant SA recovery, as maximum free SA contents (4.73 ng/g) was documented in plants that received 5 μM Kn and 100 mM NaCl (**Figure 6**). Current finding suggests that Kn stimulates free SA biosynthesis in soybean, while an addition of Kn to salt stress plants greatly mitigated the down-regulatory effect of NaCl on these plants (**Figure 6**).

### Kinetin Down-Regulate ABA Biosynthesis in Soybean

**FIGURE 7:**
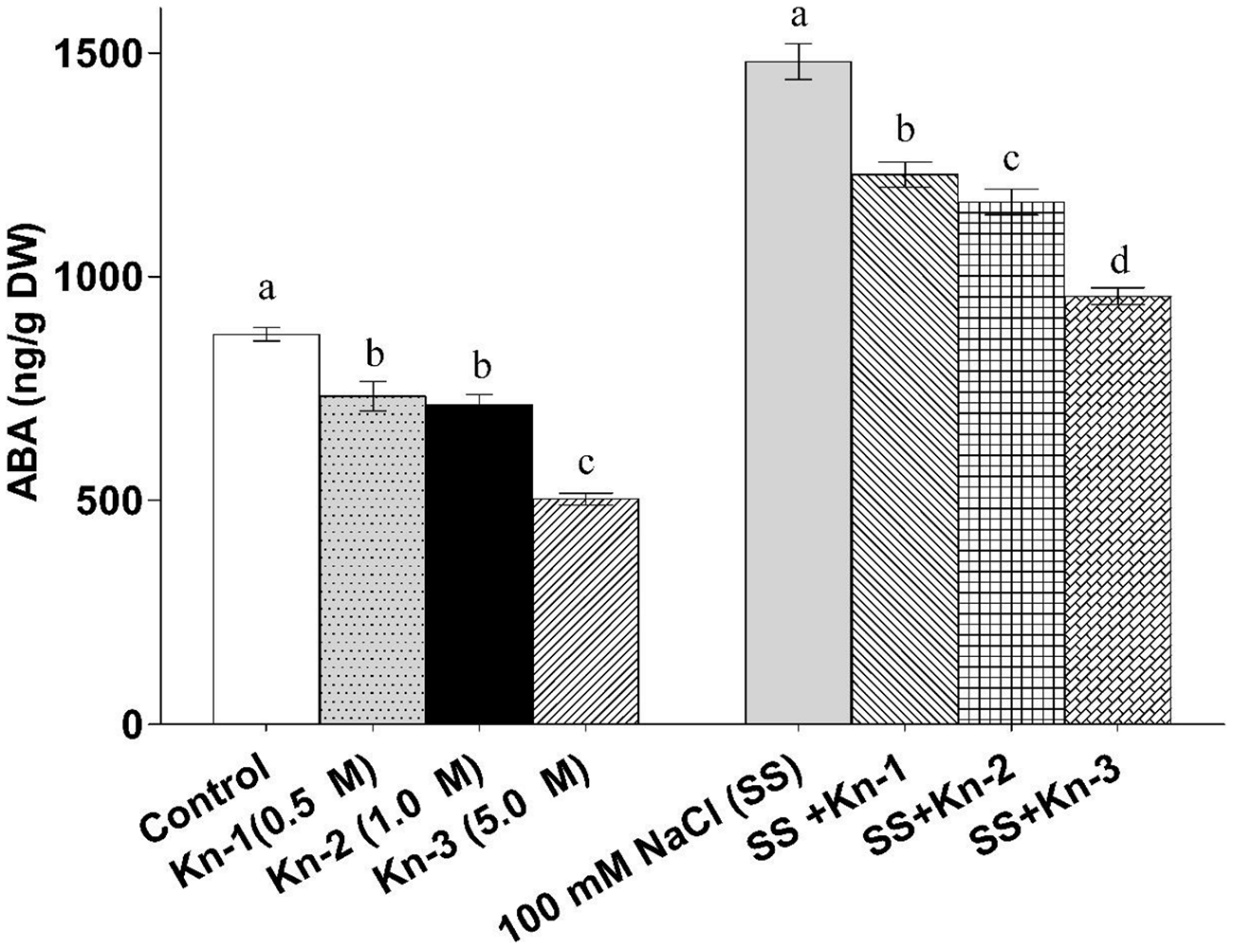
**Effect of exogenous application of Kn and NaCl on the endogenous level of Abscisic acid (ABA) of soybean shoots**. Seedlings of soybean were exposed to different concentrations of Kn and NaCl after 17 days of sowing (17 DAS) and harvested after 24 h. Graph represents data from nine replicates in two independent experiments (data are mean ± SD; *n* = 18). Bars labeled with different letters shows significant difference (*p* < 0.05; Duncan test).

Abscisic acid analysis showed that endogenous free ABA contents of soybean considerably decreased in plants treated with elevated Kn, as compared to control. Contrary to Kn, the NaCl treated plants recorded maximum ABA contents (1480.6 ng/g), while the quantity of ABA was significantly higher in treatments that received both of Kn and salinity stress as compared to sole application of Kn (**Figure 7**). Current study suggests that NaCl triggers higher ABA biosyntheses in soybean, while addition of Kn hinders such process.

### Kinetin up-Regulates Jasmonic Acid Contents but Show Antagonism Under Salt Stress

**FIGURE 8:**
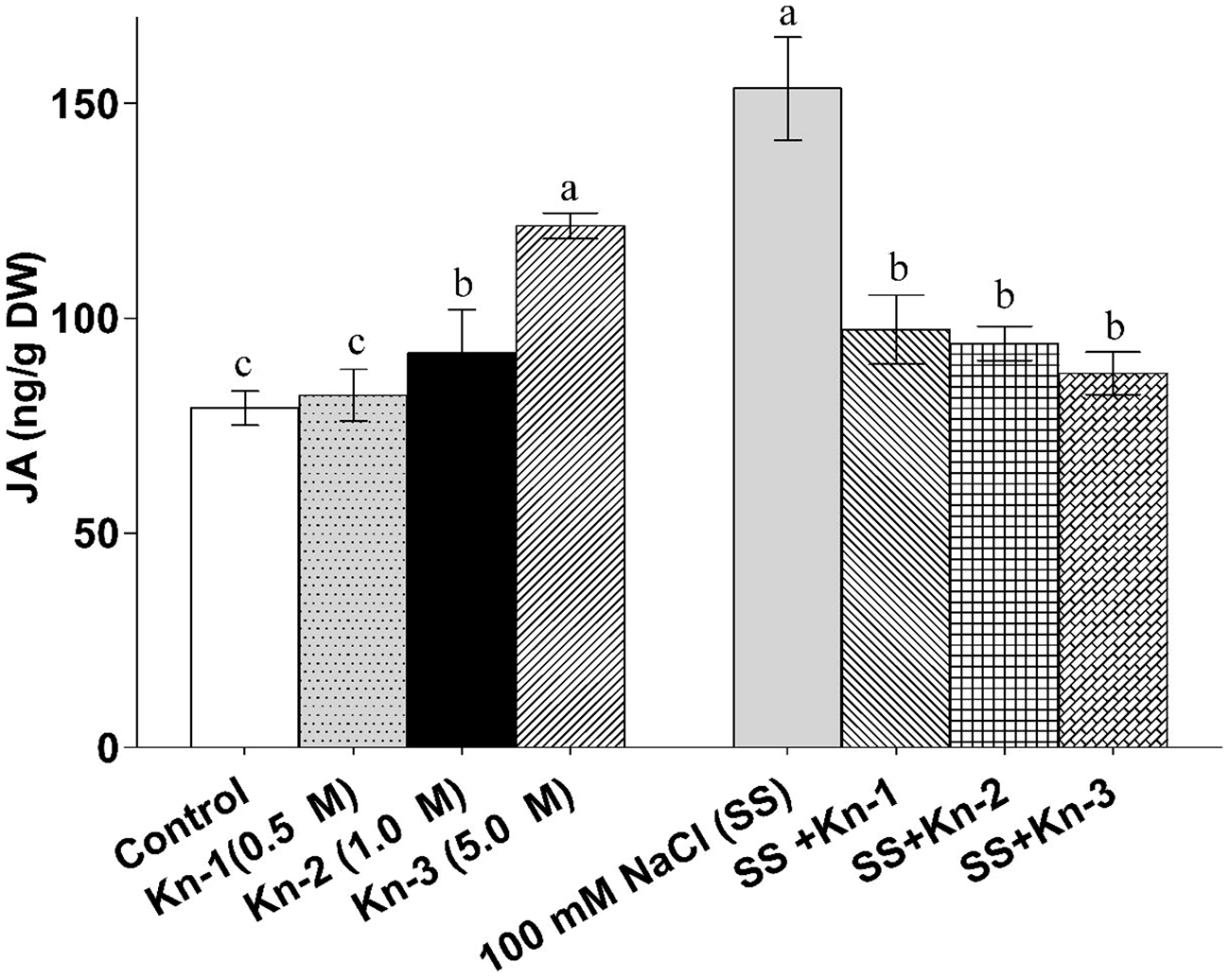
**Effect of exogenous application of Kn and NaCl on the endogenous level of Jasmonic acid (JA) of soybean shoots**. Seedlings of soybean were exposed to different concentrations of Kn and NaCl after 17 days of sowing (17 DAS) and harvested after 24 h. Graph represents data from nine replicates in two independent experiments (data are mean ± SD; *n* = 18). Bars labeled with different letters shows significant difference (*p* < 0.05; Duncan test).

Sole application of elevated Kn considerably promoted endogenous JA contents of soybean; although Kn hindered JA biosynthesis in the presence of NaCl induced salt stress (**Figure 8**). Contrary to Kn, addition of NaCl significantly up-regulated JA contents (153.47 ng/g) as compared to control (79.07 ng/g). Current study demonstrates that Kn antagonistically affect JA biosynthesis, as Kn hinders JA biosynthesis in salt stressed soybean plants.

## Discussion

Soil affected with salinity is a major limiting factor to feed human population because it interferes with crop yield and our current research findings confirm it, as growth variables of soybean drastically decreased in all treatments exposed to NaCl induced salt stress. There are multiple reasons for a declined crop growth under saline conditions. High amount of Na^+^ in soybean cell reduce its growth by making osmotic potential more negative, thereby reducing the uptake of water and minerals like K^+^ and Ca^2+^. Maintaining a low Na^+^/K^+^ ratio in cytosol is essential for normal cell because K^+^ activates more than 50 cytosolic enzymes which do not function normally under high Na^+^/K^+^ ratios (Zhu et al., 1998; Munns et al., 2006). Being physico-chemically similar, these monovalent cations compete each other for uptake by plant root. Thus, under saline conditions, greater uptake of Na^+^ results in high Na^+^/K^+^ ratios in the cytosol, which exert metabolic toxicity as they compete for the binding sites of several enzymes (Tester and Davenport, 2003). When chloroplast of soybean accumulates excessive amount of Na^+^, light driven electron flow is often significantly reduced, accompanied by inhibition of PS-II activity and reduced growth rate (Kirst, 1990; Kao et al., 2003). Low chlorophyll phenotype of soybean under elevated Na^+^ stress has been associated with enhanced ethylene production which is followed by inhibition of chlorophyll biosynthesis (Khan, 2003). The situation is further complicated by increased activity of chlorophylase in plants growing under stress (Singh and Jain, 1982). Hence, low chlorophyll content could be the result of less production and enhanced degradation of this pigment under salt stress. Contrary to the effect of salt stress on soybean, Kn significantly ameliorated the negative impact of salt stress on growth characteristics of soybean. The chlorophyll contents per unit area of soybean was little lower in Kn treated plants as compared to control, but as the leaf size was bigger in these plants, so the total chlorophyll contents could be much higher (Hamayun et al., 2010). Our current findings confirm earlier reports on the favorable role of Kn in growth and biomass of strawberry, maize, and apple (Gunes et al., 2007; Sharma and Singh, 2009).

Quality of the food can be improved by enhancing the quantities of beneficial compounds present in our food. Isoflavones are highly valued for human health, and soybean cultivars with higher isoflavones content have been developed over the past years (Phommalth et al., 2008). Our findings showed that addition of Kn significantly enhanced isoflovenes content of soybean and mitigated the negative effect of NaCl induced salt stress on their biosynthesis. Previous researches confirmed that cytokinins have been shown to cause an increase in anthocyanin accumulation in tissue culture and in parts of intact plants (Deikman and Hammer, 1995). Positive correlation has been reported in anthocyanin biosynthesis and the co-ordinated appearance of relevant enzymes including chalcone flavanone isomerase (CHI), flavanone-3-hydroxylase, phenylalanine ammonia-lyase (PAL), 3-*O*-flavonoid-glucosyltransferase and chalcone synthase (CHS; Dangelmayr et al., 1983; Gerats et al., 1983). All of these enzymes control the biosynthesis of isoflavones (Barz and Welle, 1992). As both anthocyanin and isoflavones are synthesized from phenylalanine through a common pathway sharing many enzymes including PAL, CHI, and CHS (Winkel-Shirley, 2001) advocating the enhanced accumulation of isoflavone in CKs treated plants. As an example, exogenous application of CKs (benzyladenine) was shown to induce expression of phenylalanine ammonia-lyase, catalyzing the first step of isoflavones biosynthesis converting phenylalanine to cinnamic acid (Deikman and Hammer, 1995). Cytokinins may indirectly enhance the biosynthesis of isoflavones by boosting SA production which in turn induces the expression of isoflavone synthase (IFS), inovoled in the conversion of flavones (liquiritigenin) to daizein and genistein (Subramanian et al., 2004).

**FIGURE 9:**
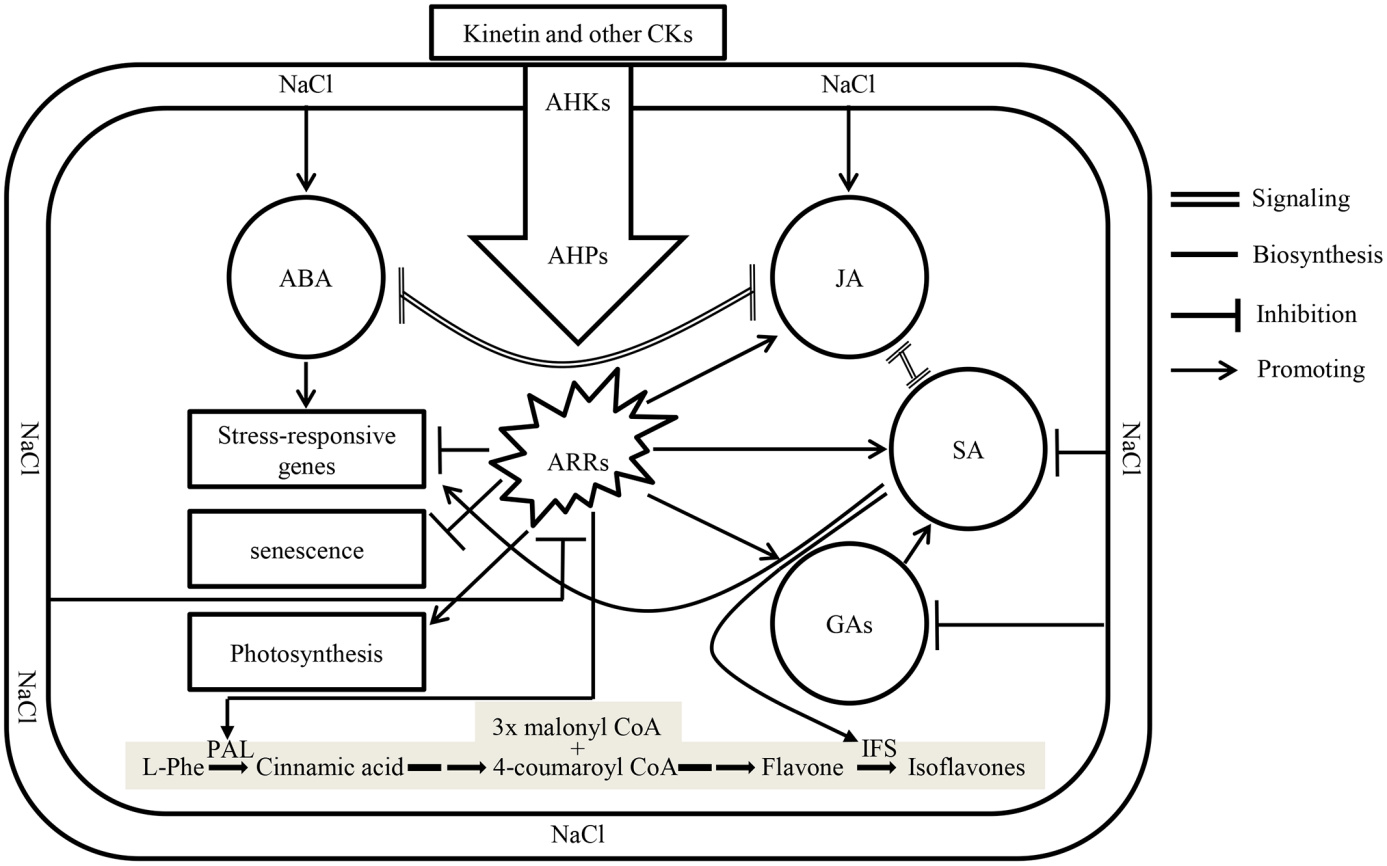
**A model showing crosstalk between different phytohormones under the influence of NaCl and exogenous Kn**. Application of salts enhances biosynthesis of ABA and JA which antagonize signaling of each other. Enhanced biosynthesis of ABA induces the expression of salt-responsive genes. Simultaneously, cytokinin (CKs) signaling, along with the biosynthesis of GAs and SA are antagonized in salt stressed plants. Application of Kn (a type of CKs) restores CKs signaling by activating transcription factors ARRs via its receptors in membrane, i.e., AHKs and downstream response regulators AHPs thereby delaying senescence, restoring photosynthesis and enhancing the biosynthesis of GAs, SA, and JA accompanied by decline in ABA concentration and signaling. The shaded pathway is indicating the biosynthesis of isoflavones in legumes, where the expression of two key enzymes, phenylalanine ammonia lyase (PAL) and isoflavone synthase (IFS) is induced by CKs and SA, respectively.

Plant growth hormones play a vital role in plant growth and development, and their responses to environmental changes are of significant importance in understanding acclimation mechanism of plants exposed to biotic and abiotic stresses. Recently it has been suggested those CKs: ABA ratios in xylem sap control stress signaling (Alvarez et al., 2008; Schachtman and Goodger, 2008). Salt stress represses the expression of isopentenyl transferase, a gene involved in the biosynthesis of CKs in ABA dependent and independent manner, thus reducing CKs content and its signaling (Nishiyama et al., 2011). Addition of Kn to the system recovered soybean seedlings from the damaging effects of salt stress by restoring CKs signaling which reduced ABA contents and delayed senescence (**Figure 9**). Contrary to its inhibitory action on ABA biosynthesis, application of exogenous Kn enhanced the contents of major phytohormones including SA, JA, and GAs among which the last two are inhibited by the application of NaCl. Beside its effect on phytohormones, Kn antagonized NaCl by enhancing photosynthesis and avoiding senescence. In current study, the levels of bioactive GA1 and GA4 considerably increased with elevated Kn, but declined under saline stress. An interactive application appears to rescue GA biosynthesis, as significantly higher levels of bioactive GAs were found in elevated Kn treated plants, as compared to sole NaCl applied plants. GA stimulates growth by promoting the destruction of DELLAs (Fu et al., 2004), while a decline in endogenous GA biosynthesis was due to the activation of growth-repressing effects of DELLAs, as salt-activated signaling pathways enhance DELLA production (Achard et al., 2006). Contrary to GA, accumulation of DELLAs prolongs the phase of vegetative growth and slows the overall rate of growth. This enhanced growth repression is distinct from passive growth rate reductions due to salt-induced perturbation of the physiological and metabolic processes that drive growth (Achard et al., 2006). In vegetative plant tissues, GA is mostly synthesized by an Early C_13_-hydroxylation pathway (Sponsel, 1987), though other pathways, especially the non-C13-hydroxylation pathways, are also often present (Zhu et al., 1991). Current findings showed the presence of both these pathways in soybean, and non-C_13_-hydroxylation that leads to the biosynthesis of GA_4_, is the major GA biosynthesis pathway in soybean.

In current study, contrary to bioactive GAs, endogenous contents of free ABA significantly enhanced in salt affected soybean but declined in plants treated with Kn alone. The endogenous ABA usually increases under drought, salinity, and cold stress (Chen and Gusta, 1983; Larosa et al., 1987; González et al., 2001). A decline in ABA level clearly suggests that addition of Kn mitigated the adverse effects of salt stress, probably by promoting plant growth and subsequently hindering build-up of DELLA proteins. ABA pathway is involved in plant responses to diverse abiotic and biotic inputs, and it is more likely that DELLA restraint provides a general mechanism for integration of plant growth responses to the environment. Unlike ABA, the JA contents increases with elevated Kn, but decreases in response to combine application of Kn and salt stress. An increase in JA content may be correlated with plant growth, which was higher in treatments. This increase in JA level can be better explained in context of vegetative storage proteins (VSP) formation, as higher VSP accumulation may have taken place under the influence of elevated Kn application (Jang et al., 2008). We observed that endogenous JA contents were significantly higher in sole NaCl treated plants as compared to control, which shows that JA could be involved in the perception of stress factors. Current study confirms previous reports about JA involvement in the perception of stress factors (Pedranzani et al., 2003), and an increase in JA contents in response to salt stress (Wang et al., 2001). Reduction in JA level under the combined influence of salt stress and Kn suggested that alleviation of salt stress by this CK is independent of JA signaling. However, in the absence exogenous CKs, JA signaling may be important for salt stress tolerance achieved by upregulating the expression of arginine decarboxylase, ribulose 1·5-bisphosphate carboxylase/oxygenase (Rubisco) activase and apoplastic invertase genes (Walia et al., 2007). Low JA phenotype of seedlings receiving both CKs and NaCl may be due to the enhanced production of SA which is known to inhibit JA signaling and therefore may indirectly reduce JA biosynthesis. High GAs in CKs treated seedlings may act synergistically with SA in reducing JA signaling thereby making plant insensitive to JA. Additionally, enhanced GAs also promote SA signaling by degrading DELLAs proteins (Navarro et al., 2008).

Salicylic acid is an important signaling substance that induces systemic acquired resistance (SAR) against pathogenic attack on plants (Siegrist et al., 2000). We observed that SA contents of soybean fractionally increased in Kn added treatments, while significantly up-regulated in plants that received both Kn and NaCl. Taking this together with reduced in SA contents of seedlings grown under salt stress alone, a positive role of SA for salt stress tolerance in soybean is suggested. Previous reports on the role of SA for salt stress have been contradictory. It has been shown that SA increases the oxidative damage generated by NaCl stress via amplifying the effects of ROS initial levels, which in turn is critical for seedling lethality under salt stress. Using mutant *Arabidopsis*, a similar role in stress response and plant-pathogen interaction has been proposed for SA, claiming that SA signaling forms a feedback amplification cycle in concert with ROS (Jabs, 1999). However, low SA mutants are not tolerant to high amount of salt, indicating that part of the oxidative stress generated during NaCl exposure is independent of the SA (Borsani et al., 2001). Contrary to this, NPR1-dependent SA signaling plays central role for salt and oxidative stress tolerance in *Arabidopsis* (Jayakannan et al., 2015).

## Conclusion

Kinetin triggers higher growth and isoflavones contents in soybean, and mitigated the adverse effects of NaCl induced salt stress. The favorable role of Kn in mitigating salt stress is evident from the levels of endogenous phytohormones, which provides an important clue for understanding the defense mechanisms of soybean against salinity. NaCl exerts adverse effect on plant growth by enhancing the production of growth inhibitory hormone ABA accompanied by a decline in the level of growth and defense related hormones including CKs, GAs, and SA. To counteract the salt stress, level of endogenous JA is enhanced which is involved in the perception of stress factors. The exogenous application of Kn oppose the effect of salt stress by reducing ABA level and promoting the production GAs and SA. Alleviation of salt stress by Kn is independent of JA signaling, indicated by the low level JA phenotype under the combined influence of salt and Kn. Kn also enhances isoflavones biosynthesis directly by inducing the expression of key enzymes of the pathway or by SA signaling which together with growth regulation, augment food quality.

## Conflict of Interest Statement

The authors declare that the research was conducted in the absence of any commercial or financial relationships that could be construed as a potential conflict of interest.
